# Westminster Hospital

**Published:** 1832-09

**Authors:** 


### WESTMINSTER HOSPITAL.

### *Phlegmasia Dolens in the Male.**

THOMAS WICKS, a sailor, twenty-three years of age, was admitted under Dr. Bright, 20th of June ult. He has led a seafaring life for nine years. His health was perfectly good until about a half year ago, when he had syphilis in a severe form, and was treated for it in the hospital at Malta. On going back to his ship he caught cold, and was obliged to return to the hospital with pain of chest, cough, and expectoration. He did not completely recover whilst in the Mediterranean, and returned to England two months since, and has been under the care of a medical man at Edmonton. He is a stout, well-formed lad, with a well-developed muscular system, and a plentiful adipose covering, but the entire surface has a pale lardaceous hue. He was treated with blisters and other medicaments whilst at Edmonton, but without much benefit. He complains now of great pain, increased on pressure, and tenderness across the upper part of the abdomen. He has had much nausea, and has vomited a considerable quantity of greenish fluid; he has also been much purged. The bowels are open at present; the tongue is furred; pulse seventy, full, and soft. Passes freely his urine, which is high-coloured; and his skin is moist and warm. A calomel and rhubarb bolus at bedtime, and an aperient dose in the morning. A saline mixture every four hours.

23d. The pain and tenderness of abdomen have abated; the tongue is slightly furred; the bowels freely open; no nausea; pulse natural.

26th. The pain of bowels has entirely subsided; a dull ache is perceived across the loins. The chest is perfectly healthy; tongue

* The Lancet.

clean; bowels open; micturition free; pulse eighty-two, feeble; appetite reviving: continues the saline mixture.

28th. Great tumefaction; pain and tenderness have rapidly come on in the left thigh and leg. The tumefaction is general over the limb, but the pain and soreness are most marked over the course of the femoral vessels. Pulse ninety; bowels open; tongue furred. To be cupped to eight ounces on the inside of the thigh, and to continue the saline medicine.

29th. The patient declares himself relieved by the abstraction of blood; the swelling of the limb is less, but the pain of thigh is undiminished. The whole limb is extremely tense and sore, and the leg is a little œdematose; pulse full; tongue clean; bowels open. Venesection to twelve ounces, and two tablespoonfuls of the following mixture to be swallowed every four hours: Take of Antimonial wine, one ounce; Sulphate of magnesia, one ounce; Camphor julep, six ounces. Mix.

Fomentations of poppy-heads to be assiduously applied.

30th. The blood drawn from the arm is neither buffed nor cupped. The tumefaction and tension have abated, but the pain is undiminished. Pulse eighty-six, full, soft, and regular; tongue clean, and bowels open.

July 1st. The pain is relieved by the fomentation. The limb is still swollen; the pain is most acute in the groin, extending upwards along the iliac, and downwards along the femoral vessels; pulse ninety-eight. Continues the remedies.

2d. Has still pain, especially on moving the limb; he cannot bear pressure over any part of the femoral vein; tongue furred; bowels open; secretion of urine diminished; pulse 100, rather hard; countenance anxious: twenty leeches to be applied to the groin, and subsequently fomentations.

3d. Pain not so severe; swelling less; the pain is principally felt in the groin, and in the ham; secretion of urine scanty; pulse 102, feeble; countenance less anxious; bowels open.

4th. The pain greatly increased along the course of femoral vessels last night, and was relieved by the application of twenty leeches. There are still pain and soreness of thigh, and particularly in the popliteal space; this last is augmented by any attempt to straighten the limb; tongue flabby, exsanguous, and slightly furred; pulse 100, feeble.

6th. No very material alteration. There is no great difference in the size of the two limbs, except in the circumference of the upper part of the thigh, where the affected limb is by much the larger, and at the ankle, where a relative difference of size exists; bowels open thrice daily; stools liquid; urine more copious, and of lighter colour; pulse 104, regular, feeble. Twenty-four leeches to be placed on the inside of the affected thigh.

8th. The limb is much the same in appearance. Twenty-four more leeches applied.

9th. The patient imagines the pain is increased by the use of fomentations. An evaporating spirit lotion is, consequently, substituted.

12th. Pain and swelling nearly in *statu quo*. The redness and tension are perhaps a little diminished; the foot and ankle are œdematose; tongue pallid and flabby; bowels freely moved; pulse 100, tolerably full.

14. The pain, redness, and tumefaction, have rapidly diminished since the use of the spirit lotion. Tongue clean; bowels open; pulse eighty-eight.

17th. The limb is perfectly easy whilst the patient remains quiet, but on the slightest attempt at motion, the pain returns as severely as ever. Tongue clean, but flabby; bowels open; pulse eighty, full.

18th. The patient was seized with rigors about an hour ago, and still continues shivering. The limb, too, has rapidly swollen since that time, particularly in the groin, and he cannot bear the slightest touch; tongue furred; bowels open once; pulse 120: twelve leeches to the groin; ten grains of Dover's powder exhibited immediately.

Eight o'clock P.M. Pain relieved by the leeches, but the swelling remained unreduced; the pain, however, returning as severe as formerly. Venesection to sixteen ounces was had recourse to, and proved effectual in removing the pain.

19th. The limb has remained easy since the venesection, but the intumescence has rather increased. Erysipelas has made its appearance in the leg from the foot to the knee, and appears to be occasioned by the leech-bites. Tongue pallid; bowels open once; pulse 102, full and hard. A strong purging draught with alkali to be given; to continue the antimonial mixture.

20th. The erysipelas is subsiding; swelling of groin smaller; pain less severe; tongue furred; pulse eighty, full and soft; countenance cheerful. To repeat the alkaline purge.

23d. The swelling in the thigh, and the erysipelas in the leg, are gradually disappearing; tongue clean, but flabby; bowels open; pulse eighty-four, full. The patient can move the limb without pain. He sleeps well, and his appetite is reviving.

24th. A resuscitation of the erysipelas on the dorsum pedis; bowels constipated; the thigh is still rather tumefied, but there is no pain. Six leeches to be applied to the foot, and an alkaline purging draught to be swallowed immediately.

Aug. 3d. The erysipelas has nearly gone; the tumefaction still unremoved; the pain and inflammation have subsided; the emunctories apparently act well, but the *ensemble* of the patient presents an unhealthy leucophlegmatic character.

10th. The patient's general condition has improved; his appetite is good; the pain and tumefaction of thigh-bone abated, and are scarcely appreciable, but the œdema of the leg has very much augmented. The use of the evaporating lotion is continued, and occasional purges are administered; his colour is slightly improved.

This is a case somewhat analogous to those described by Sir Henry Halford at one of the evening *converzatione* in the College of Physicians. The exciting cause, viz. the sudden application of cold, was the same, but in the present case no remarkable slowness, or intermission, of pulse, has been observed, probably because no obliteration of the veins has taken place, although a suspicion of such a fact is excited by the increased œdema of the affected, and a certain hardness perceptible in the course of the femoral vessels. This is a circumstance, however, which remains to be proved by the subsequent history of the case.

